# Appraisal on the indications, subtypes, and complications of surgically treated thyroid diseases for thyroidologists: A systematic review and meta-analysis in thyroidology

**DOI:** 10.1016/j.sopen.2025.12.005

**Published:** 2026-01-06

**Authors:** Ilker Sengul, Demet Sengul

**Affiliations:** a Thyroidology Unit, Giresun University Faculty of Medicine, TR28100, Giresun, Turkey; b Division of Endocrine Surgery, Giresun University Faculty of Medicine, TR28100, Giresun, Turkey; c Department of General Surgery, Giresun University Faculty of Medicine, TR28100, Giresun, Turkey; d Department of Pathology, Giresun University Faculty of Medicine, TR28100, Giresun, Turkey

**Keywords:** Thyroid gland, Subtype, Surgery, Pathology, Thyroidology, Thyroidologists

Dear Editor

We address this letter to offer a brief, albeit essential, critique upon the recent systematic review and meta-analysis submitted by Getachew et al. [1], concerning the indications, sub-types, and attendant sequelae following surgical intervention for thyroid pathologies within the low and middle-income African nations, published in volume 27, *Surgery Open Science*. The scholarly diligence exhibited in collating data hitherto dispersed merits recognition, particularly as it illuminates a pooled prevalence of postoperative complications which doth stand notably higher when compared with established international figures in thyroidology [2], [3], [4], [5], [6], [7], [8]. Yet, the necessity for a measured interpretation of the statistical conclusions remains paramount. The analysis reveals that toxic goiters constitute the most frequent indication for thyroidectomy (46.62 %), closely followed by cosmetic considerations (41.07 %). Notwithstanding the import of these figures, the inherent methodology is beholden to a profound statistical heterogeneity (I^2^ values reaching 99.9 % for both toxic goiter and cosmetic rationale). Furthermore, the authors' detection of significant publication bias, particularly regarding the toxic goiter indication, calls for the utmost circumspection when one seeks to generalize the resultant pooled prevalence data. Of note, it is further observed that the practice of subtotal thyroidectomy remains the predominant procedure within this geographical remit (37.28 %), a finding that departs noticeably from the predilection for near-total or total resections commonly observed in more developed jurisdictions. Furthermore, the researchers postulate that this adherence to subtotal resection may stem from the manifold difficulties encountered in providing rigorous postoperative monitoring and continuous hormonal replacement therapy within these facilities. Despite the conservative nature of this surgical approach, the pooled rate of complications is elevated at 26.6 %. Moreover, the incidence of hypoparathyroidism and recurrent laryngeal nerve injury might be an alarming statistics that merit immediate redress. One must heartily endorse the final recommendations: that all concerned parties collaborate to establish management protocols, provide logistical support, and furnish specialized training for general surgeons to raise their practice to the standard of endocrine surgery, whose findings, even amidst the constraints of heterogeneous data and variations in reporting, serve as a clear and imperative mandate for clinical amelioration for thyroidologists. This issue merits further investigation. We thank Getachew et al. [1] for their study in *Surgery Open Science*.

## CRediT authorship contribution statement

**Ilker Sengul:** Writing – review & editing, Writing – original draft, Visualization, Validation, Software, Resources, Project administration, Methodology, Investigation, Formal analysis, Conceptualization. **Demet Sengul:** Writing – review & editing, Writing – original draft, Visualization, Validation, Supervision, Software, Resources, Project administration, Methodology, Investigation, Formal analysis, Conceptualization.

## Consent to participate

Not applicable.

## Consent to publish

Not applicable.

## Ethics approval

Not applicable.

## Code availability

Not applicable.

## Funding

None declared.

## Declaration of competing interest

The authors declare no competing interests.

## Data Availability

Data sharing is not applicable to this article as no new data were created in this study.
